# Spatio-temporal visualization and forecasting of ${\text {PM}}_{10}$ in the Brazilian state of Minas Gerais

**DOI:** 10.1038/s41598-023-30365-w

**Published:** 2023-02-25

**Authors:** Kim Leone Souza da Silva, Javier Linkolk López-Gonzales, Josue E. Turpo-Chaparro, Esteban Tocto-Cano, Paulo Canas Rodrigues

**Affiliations:** 1 Department of Statistics, Federal University of Bahia, Salvador, Brazil; 2 UPG Ingeniería y Arquitectura, Escuela de Posgrado, Universidad Peruana Unión, Lima, Peru; 3 Facultad de Ingeniería y Arquitectura, Universidad Peruana Unión, Lima, Peru

**Keywords:** Ecology, Environmental sciences

## Abstract

Air pollution due to air contamination by gases, liquids, and solid particles in suspension, is a great environmental and public health concern nowadays. An important type of air pollution is particulate matter with a diameter of 10 microns or less (${\text {PM}}_{10}$) because one of the determining factors that affect human health is the size of particles in the atmosphere due to the degree of permanence and penetration they have in the respiratory system. Therefore, it is extremely interesting to monitor and understand the behavior of ${\text {PM}}_{10}$ concentrations so that they do not exceed the established critical levels. In this work, we will study the ${\text {PM}}_{10}$ concentrations in all available monitoring stations in the Brazilian state of Minas Gerais. To better understand its behavior, we will provide a spatio-temporal visualization of the ${\text {PM}}_{10}$ concentrations. Besides the descriptive and visualization analysis, we consider six standard and advanced time series models that will be used to fit and forecast ${\text {PM}}_{10}$ concentrations, with application to three locations, one in Belo Horizonte, the Minas Gerais state capital, and the monitoring stations with the lowest and highest average ${\text {PM}}_{10}$ concentration levels.

## Introduction

The human impact on the planet is remarkable, and the attempt to reduce these impacts is increasingly urgent. Air pollution, for example, is directly related to the environment and human health^1–3^. One of the determining factors that affect human health is the size of particles in the atmosphere due to the degree of permanence and penetration they have in the respiratory system^4–7^. As the health impact is directly related to the particle size, monitoring the ${\text {PM}}_{10}$ concentrations, particulate materials smaller than or equal to 10 micrometers, is very important^8,9^. Based on the annual average of ${\text {PM}}_{10}$, the World Health Organization (WHO) ranked Ahvaz in Iran as the most polluted city in the world at 372 $\upmu$g/m$^3$^10^.

In this scenario, in Europe, the Apheis project has developed guidelines for analyzing and collecting data on air quality, and public health impacts^11^. The study presented the health impact in 19 Eastern and Western European cities. The results indicate that reducing long-term ${\text {PM}}_{10}$ exposure by 5 $\upmu$g/m$^3$ could prevent approximately 3300–7700 premature deaths annually. The Apheis project also showed that in urban Europe, current air pollution has a non-negligible impact on public health and that even in cities with low air pollution, preventive measures can reduce damage^12^. For its part, in Brazil, in the metropolitan region of São Paulo (MASP), 40% of ${\text {PM}}_{10}$ emissions come from mobile sources^13–15^. In addition, ozone and ${\text {PM}}_{10}$ are the pollutants with the greatest impact on air quality at MASP^16,17^. A study carried out in the Jânio Quadros and Maria Maluf tunnels in São Paulo indicates that the emission of heavy diesel vehicles is the major source of ${\text {PM}}_{2.5}$ fine particulate matter^14^. Likewise, a study in the metropolitan region of Lima, the capital of Peru, proposed a space-time visualization to analyze ${\text {PM}}_{10}$ levels, showing that the highest concentrations of ${\text {PM}}_{10}$ were recorded near hills and high-traffic roads and unpaved streets^18^.

In particular, in Brazil, through Conama Resolution No. 005/1989, the National Air Quality Control Program (Pronar) was created^19^. This program attempts to build the foundations for a national air quality protection policy^20^. However, although Pronar is the beginning of a national air quality policy, it has great legal fragility because its legal basis is hierarchically inferior to the already established laws. Furthermore, there is a clear asymmetry between the country’s regions and most of the air quality management instruments are located in southeast Brazil^21^.

Several studies with air pollution data were developed with this challenge, using statistical models to address both model fit^22–24^, and model forecast^25–27^ of air pollution in Brazil. For example, in Itabira (a city in the Brazilian state of Minas Gerais), an increase of 10 $\upmu$g/m$^3$ of ${\text {PM}}_{10}$ was associated with an increase in respiratory diseases in the emergency room, concluding that an increase in ${\text {PM}}_{10}$ levels has a major impact on the exposed population^28^. Likewise, a study carried out in the Greater Vitória Region (the capital of the Brazilian state of Espírito Santo) used the seasonal auto-regressive integrated moving average with exogenous factors (SARIMAX) model to better understand and predict the behavior of ${\text {PM}}_{10}$ concentrations, noting that both wind speed and rainfall were statistically significant and helped to improve the model fit^29^. On the other hand, in a study carried out in the Brazilian State of Rio Grande do Sul, it was presented that the auto-regressive integrated moving average with exogenous factors (ARMAX) model, with the inclusion of the exogenous variables (Carbon Monoxide and Sulfur Dioxide), obtained better performance when compared to the autoregressive integrated moving average (ARIMA), simple exponential smoothing, and Holt-Winters models, for ${\text {PM}}_{10}$ prediction^29^.

Meanwhile, other methods for time series forecasting, including neural networks^30–33^ and deep learning have also been used to forecast variables related to air pollution. For example, one study proposed predictive models for ${\text {PM}}_{2.5}$ concentration with a model that combined the fast Fourier transform and long short-term memory neural network (FFT-LSTM) and proved to be superior to the traditional LSTM and extended long short-term memory recurrent neural network (LSTM) models^34^. Another study used a deep learning algorithm integrating convolutional neural networks (CNNs) and LSTM neural networks to predict ${\text {PM}}_{2.5}$ concentrations^35^. Cordova et al.^36^ studied the spatio-temporal behavior of air quality in Metropolitan Lima, evaluated and predicted the ${\text {PM}}_{10}$ concentrations using the recurrent artificial neural network LSTM, based on the past values of this pollutant and three meteorological variables obtained from five monitoring stations. It is important to notice that the ${\text {PM}}_{10}$ concentrations have nonlinear behavior and fluctuate strongly in spatio-temporal scales^37^ due to the nonlinear character of the atmospheric wind speed^38^. Consequently, to manage this strong variability, in this paper, we consider different forecasting models.

Although many studies have been made to better understand the behavior and to forecast ${\text {PM}}_{10}$ concentrations, no comprehensive study that includes all monitoring stations in the Brazilian state of Minas Gerais has been made. In this paper, we will analyze the spatio-temporal dynamics of ${\text {PM}}_{10}$ concentrations in all available monitoring stations in the Brazilian state of Minas Gerais. Then, we compare classical parametric models and neural networks to forecast the ${\text {PM}}_{10}$ concentrations, whose results can be useful for governmental agencies and policymakers to decide on specific policies and actions to improve air quality.

The rest of the paper is structured as follows. The following section describes the data collection, data cleaning, and the methods and models used for ${\text {PM}}_{10}$ forecasting. The section “Results and discussion” presents the descriptive analysis and the main findings of this research regarding model fit and model forecasting. Finally, the section “Conclusions” provides the main conclusions of this paper, together with some recommendations for future research.

## Materials and methods

### Data collection and data cleaning

The data used in this work was collected by the State Foundation for the Environment of the Brazilian state of Minas Gerais. The data was collected hourly and the last five years available were considered (between 2015 and 2019) in all 58 monitoring stations. The data are publicly available per municipality, monitoring station, and year. For each combination municipality/monitoring station/year, the data is available in a csv file that includes the hourly information on pollutant levels such as ${\text {PM}}_{10}$ and ${\text {PM}}_{2.5}$, as well as meteorological data such as temperature, wind direction, rainfall, atmospheric pressure, wind speed, radiation, and relative humidity. The first step of the analysis was to organize, clean, and store the database, which is always a challenging operation when dealing with real data. In this case, the main challenges were:

- The lack of information for some variables in several stations.
- A significant amount of missing values.

**Figure 1:**
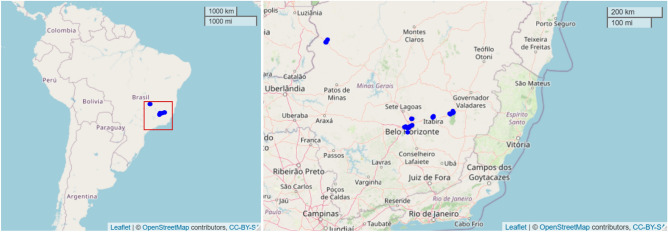
Map of South America (left) and map with the location of the monitoring stations (right). The source map was made with the R package leaflet, version 2.1.1.

From the available 58 monitoring stations, we decided to discard those with a percentage of missing values above $35\%$ in the ${\text {PM}}_{10}$ data. In addition, one station that did not have data for 2015 was also discarded. Thus, we proceeded with data from 29 air quality monitoring stations distributed throughout the Brazilian state of Minas Gerais. The locations of the 29 monitoring stations considered in this study can be seen in Fig. 1, with more stations in areas with higher population density, resulting in some overlapped points in the map. Figure 6S of the “Supplementary material” shows a heat map of the missing ${\text {PM}}_{10}$ values in each monitoring station, and Table 1S gives the rate of the missing values. The next stage was the imputation of missing values, which was done by using the function na_kalman of the package imputeTS in the R software^39^. At the end of the process, we obtain a database of ${\text {PM}}_{10}$ concentrations with 43824 hourly observations (rows) for each of the 29 stations (columns) available in the “Supplementary material”. Table 1S of the “Supplementary material”, presents detailed information for each monitoring station, including code, station name, company responsible for the monitoring station, longitude, latitude, and the rate of missing values.

### Models for time series forecasting

Time series models are very important and can be useful in many areas of knowledge that collect time-dependent data^40,41^. They can be used both to understand the underline process that generated the data and to predict future observations^42,43^. Predictions can be for a short term (e.g., 1 h ahead) and for a long term (e.g., 720 h–1 month ahead). Despite the forecasting horizon, forecasting is an important aid to effective planning, and policy-making^44^. In this study, six models for time series forecasting of ${\text {PM}}_{10}$ levels are considered and briefly described in the sequence.

#### Seasonal Naive

The Seasonal Naive (SNAIVE) model is an extension of the NAIVE model that considers a seasonal component of period *T* in the time series^45^ and can be written as

1 $$\begin{aligned} \widehat{Y}(t+h|t) = Y(t+h-T), \end{aligned}$$

where *t* is the length of the time series, *h* is the forecasting horizon, *T* is the seasonal period, $\widehat{Y}(t+h|t)$ is the prediction *h* steps ahead, and $Y(t+h-T)$ is the observed value *T* observations before the length of the series, *t*, minus the forecasting horizon, *h*. This means that the seasonal naive model estimates the out-of-sample forecast as the last observation at the same seasonal point. When considering $T=1$, the NAIVE model is obtained. This model was adjusted using the snaive function of the package forecast in the software R.

#### Seasonal Naive + Decomposition

Let us consider the three-part decomposition of the time series *Y*(*t*) of length *t*,

2 $$\begin{aligned} Y(t) = T(t) + S(t)+R(t), \end{aligned}$$

where *T*(*t*) is the trend of the time series, *S*(*t*) is the seasonal component, and *R*(*t*) is the rest/residual of the time series. Although several techniques are available to estimate the components in the decomposition, we consider the STL (Seasonal and Trend decomposition using Loess) for its versatility and robustness. The model Seasonal Naive + decomposition firstly removes the seasonality *S*(*t*) of the time series *Y*(*Y*),

3 $$\begin{aligned} \widehat{Y}(t) = Y(t)-S(t), \end{aligned}$$

and then uses the NAIVE model to forecast the time series with the seasonal adjustment, which is added to the seasonal adjustment of the last time period of the time series to obtain the final forecast. The decomposition and forecasts can be obtained by using the stl and naive functions of the R software.

#### Exponential Smoothing + Decomposition

Exponential smoothing is one of the most used and well-known methods for time series forecasting^46^. The forecast *h* steps ahead for the simple exponential smoothing can be written as:

4 $$\begin{aligned} \widehat{Y}(t+h|t) = \alpha y(t) + \alpha (1-\alpha ) y(t-1) + \alpha (1-\alpha )^2 y(t-2) + \cdots , \end{aligned}$$

with $\alpha \in [0,1]$. In this way, the forecasts are obtained as a weighted average of past observations, with the weights decreasing exponentially as we go back in time. Various versions of exponential smoothing have been proposed to deal with trends and seasonality in time series. In this work, we use the exponential smoothing model automatically selected for the seasonally adjusted series. Further details about exponential smoothing algorithms can be found in^46^.

#### SARIMA

The seasonal autoregressive integrated moving average (SARIMA) models are among the most widely used methods for time series forecasting. They are an extension of the autoregressive integrated moving average (ARIMA) model that adds a linear combination of seasonal values and/or forecast errors. Let *Y*(*t*) be a time series. The $SARIMA(p,d,q)(P,D,Q)_s$ model can be written as

5 $$\begin{aligned} (1-B)^d(1-B^s)^D\Phi (B^s)\phi (B)Y(t)=\Theta (B^s)\theta (B)\varepsilon (t) \end{aligned}$$

where *B* is the lag operator given by $B^k=Y(t-k)/Y(t)$, $\Phi (B) = 1 - \phi _1B^1 - \phi _2B^2 z \dots - \phi _pB^p$ is an autoregressive (AR) polynomial function of order *p* with vector of coefficients $\Phi '=[\phi _1,\phi _2,\dots , \phi _p]$, $\Theta (B)=1+\theta _1B^1+\theta _2B^2+\dots +\theta _qB^q$ is a moving average (MA) polynomial of order *q* with vector of coefficients $\Theta '=[\theta _1,\theta _2,\dots , \theta _q]$, $\Phi (B^s)=1-\phi _{s,1}B^s-\phi _{s,2}B^{2s}-\dots -\phi _{s,p}B^{ps}$ and $\Theta (B^s)=1+\theta _{s,1}B^s-\theta _{s,2}B^{2s}-\dots -\theta _{s,q}B^{qs}$ are seasonal polynomial functions of order *P* and *Q*, respectively, that satisfy the stationarity and invertibility conditions, *d* is the number of differences needed to stationarize the series, *D* is the number of seasonal differences and $\varepsilon (t)$ is white noise, defined as a sequence of uncorrelated random variables with zero mean and constant variance over time, $\varepsilon _t \sim RB( 0, \sigma ^2_\varepsilon )$. The parameter estimates of the SARIMA model can be obtained with the arima function of the R software.

#### NNETAR and NNETAR + Decomposition

The Neural Network AutoRegression (NNETAR) model is an artificial neural network (ANN). ANNs are mathematical models based on the behavior of the brain that allow for complex nonlinear relationships between the response variable and its predictors^44^. A neural network comprises an input, output, and hidden layers. In the hidden layers, we find the weights ($W_i$), bias (*b*), and the activation function, which help to convert the input data into the expected output. The weights are the parameters that will determine the intensity with which each neuron affects the other. On the other hand, bias is a parameter used to adjust the output along with the weighted sum of the neuron’s inputs. In each neuron, there will be an activation process through the *z* function^47^. This process is illustrated by Eq. (6):

6 $$\begin{aligned} z = \sum _{i = 1} ^ k W_i X + b \end{aligned}$$

The forecasts using the NNETAR model and the NNETAR in the seasonally adjusted time series using the STL decomposition can be obtained with the nnetar function of the forecast package in the R software. The model receives the last observations up to time *t* and performs the forecast for time $t+1$. To obtain more predictions, the same process is repeated iteratively.

### Accuracy measures

To evaluate the performance of the models, two types of accuracy measures will be considered, one for the model fit (using the training data) and another for the model forecast (using the train set). Two accuracy measures will be used. Equation (7) defines the root mean squared error (RMSE) and Eq. (8) defines the symmetric mean absolute percent error (SMAPE). In contrast to the mean absolute percentage error, the SMAPE provides a value with upper and lower bounds, with values between zero and one.

7 $$\begin{aligned} \text {RMSE}= & {} \sqrt{\frac{1}{n} \sum _ {i=1} ^ n (y_i - \widehat{y}_i)^2} \end{aligned}$$

8 $$\begin{aligned} \text {SMAPE}= & {} \frac{100\%}{n} \sum _{i = 1} ^ n \frac{|y_i - \widehat{y}_i|}{|\widehat{y}_i|+|y_i|} \end{aligned}$$

In both equations, *n* is the number of observations (i.e., length of the train or test data), $y_i$, $i=1,\dots ,n$ are the observed real values, and $\widehat{y}_i$ are the estimated or forecast values.

## Results and discussion

### Descriptive analysis

**Figure 2:**
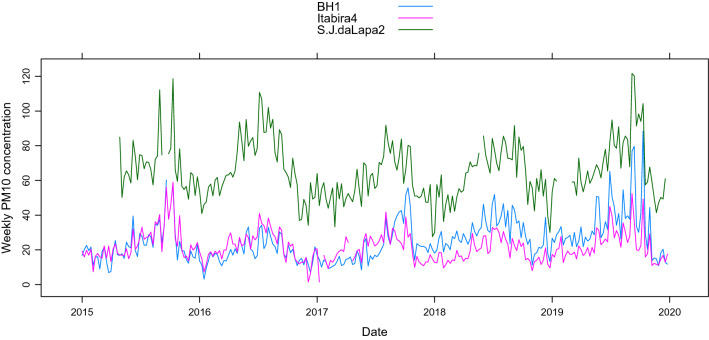
Average weekly concentration of ${\text {PM}}_{10}$ (in $\upmu$g/m$^3$) between 2015 and 2019 for one monitoring station located in the Brazilian state capital of Minas Gerais, Belo Horizonte (BH1, blue), the monitoring station with the lowest average of ${\text {PM}}_{10}$ concentrations (Itabira4, pink), and the monitoring station with the highest average of ${\text {PM}}_{10}$ concentrations (S.J.daLapa2, green).

**Figure 3:**
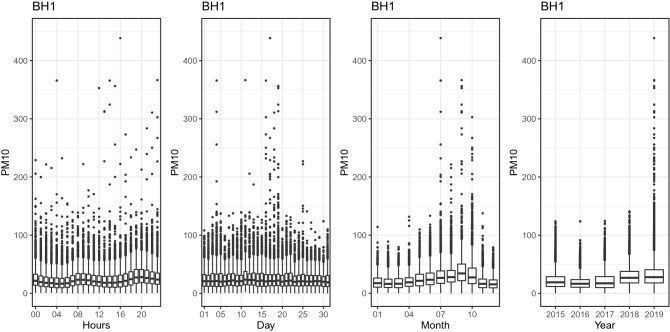
Hourly, daily, monthly and yearly boxplots for the monitoring station located in the Brazilian state capital of Minas Gerais, Belo Horizonte (BH1), respectively.

**Figure 4:**
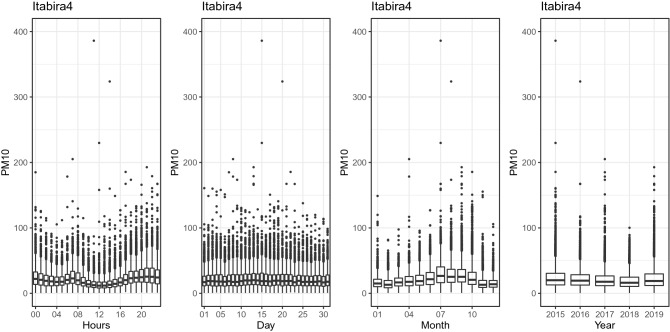
Hourly, daily, monthly and yearly boxplots for the monitoring station with the lowest average of ${\text {PM}}_{10}$ concentration (Itabira4), respectively.

**Figure 5:**
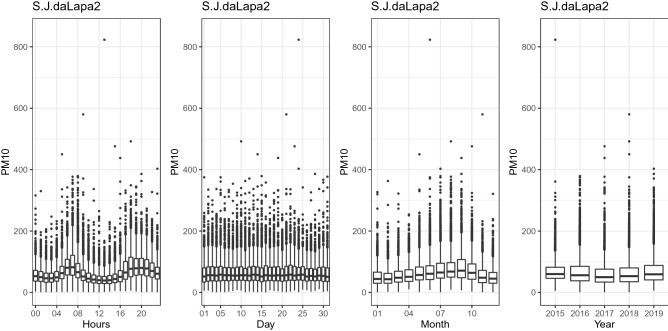
Hourly, daily, monthly and yearly boxplots for the monitoring station with the highest average of ${\text {PM}}_{10}$ concentration (S.JdaLapa2), respectively.

The database includes 43824 hourly observations (5 y between 2015 and 2019) of ${\text {PM}}_{10}$ concentrations in 29 monitoring stations in the Brazilian state of Minas Gerais. Being a large dataset results in a big challenge for its visualization. To better visualize and understand the behavior and patterns of the data, several strategies were used. The weekly average in each monitoring station is presented in Fig. 1S of the “Supplementary material”. In addition, boxplots per hour of the day, per day of the month, per month of the year, and per year are also presented in Figs. 2S–5S of the “Supplementary material”, respectively. In these plots, specific trends and patterns are visible, particularly, along the day, along the months, and along the years.

To present further results, without doing an exhaustive analysis, three monitoring stations were selected. The first is located in Belo Horizonte (BH1), the state capital city and the most populous city in the state, with its main sources of atmospheric pollution being traffic and industry. To consider the full range of the observed data in the 29 monitoring stations, the other two monitoring stations that were selected are those with the lowest (Itabira4) and the highest (S.J.daLapa2) average concentration of ${\text {PM}}_{10}$ among the available stations. Figure 2 shows the weekly average behavior of these three monitoring stations. It is possible to notice that the concentrations of BH1 and Itabira4 are very similar, with emphasis on the year 2019, where the BH1 station shows a significant increase in the average weekly concentration of ${\text {PM}}_{10}$. Among the 29 considered monitoring stations, BH1 and Itabira4 are among those with the lowest average pollution levels. São José da Lapa (S.J.daLapa2), located north of the metropolitan region of Belo Horizonte, has ${\text {PM}}_{10}$ concentrations well above the weekly average of the other two stations, which is likely due to lime and crushed stone factories located in the region. The average concentration of ${\text {PM}}_{10}$ in S.J.daLapa2 is 49.9 $\upmu$g/m$^3$ against 25.37 $\upmu$g/m$^3$ and 22.13 $\upmu$g/m$^3$ in BH1 and Itabira4, respectively.

Figure 3 shows the behavior of the hourly, daily, monthly, and annual concentration of ${\text {PM}}_{10}$ at the BH1 station. The hourly plot shows a higher concentration between 7 and 10 a.m. and between 6 and 10 p.m. In the monthly plot, higher concentrations of ${\text {PM}}_{10}$ are observed between June and October. There is also an increase in concentrations in the years 2018 and 2019. Figure 4 shows the behavior of the hourly, daily, monthly, and annual concentration of ${\text {PM}}_{10}$ at the Itabira4 station. The hourly graph shows a higher concentration between 6 and 9 a.m. and at the end of the day between 6 and 11 p.m. In the monthly plot, higher ${\text {PM}}_{10}$ concentrations are observed between June and October. Figure 5 shows the behavior of the hourly, daily, monthly, and annual concentration of ${\text {PM}}_{10}$ at the S.J.daLapa2 station. The hourly graph shows a higher concentration between 6 and 9 a.m. and between 5 and 11 p.m. In the monthly plot, higher concentrations of ${\text {PM}}_{10}$ are also observed between June and October.

All boxplots for the hourly, daily, monthly, and annual behavior of the 29 monitoring stations can be seen in Figs. 2S–5S, of the “Supplementary material”, respectively.

### Model fit

The six models defined above were used for model fit, considering the data from the three monitoring stations described in the previous subsection (BH1, Itabira 4, and S.J.daLapa2). Table 1 shows the results of the two accuracy measures, RMSE and SMAPE, for the model fit of each model, in the data from the three monitoring stations. Based on the RMSE, the best fit was obtained by the model NNETAR for the Itabira4 and S.J.daLapa2 monitoring stations, while the best model for BH1 was the NNETAR+Decomposition. When considering the SMAPE, the results for Tabira4 and S.J.daLapa2 do not change, but for BH1, the best model was the Naive+Decomposition.

**Table 1:** Accuracy measures (RMSE and SMAPE) for the model fit of each of the five considered models, in the data from the three monitoring stations, BH1, Itabira4, and S.J.daLapa2.

	BH1		Itabira4		S.J.daLapa2	
	RMSE	SMAPE	RMSE	SMAPE	RMSE	SMAPE
Seasonal Naive	18.53	0.47	14.73	0.43	39.58	0.38
Naive + Dec	8.94	0.23	8.78	0.25	29.92	0.27
Exp Smoothing + Dec	8.75	0.27	8.61	0.25	28.97	0.27
SARIMA	8.66	0.26	8.36	0.26	27.14	0.28
NNETAR	8.49	0.27	8.15	0.24	26.16	0.26
NNETAR + Dec	8.46	0.27	8.21	0.24	26.78	0.26

### Model forecasting

A similar procedure for model fit, now considering the test data, was done for the model forecast. The same six models were used, considering the data from the three monitoring stations. Table 2 shows the results of the two accuracy measures, RMSE and SMAPE, for the forecasts using each of the six models for the data from the three monitoring stations, BH1, Itabira4, and S.J.daLapa2. The accuracy measures were obtained by considering the last 14 days (336 observations) of each time series as test data. From the analysis of Table 2, it can be seen that the best model to forecast the ${\text {PM}}_{10}$ concentrations in BH1 is SARIMA. For the monitoring station with the highest ${\text {PM}}_{10}$ average, S.J.daLapa2, the Exponential Smoothing + Decomposition was the best forecasting model. On the other hand, for Itabira4, the best forecasting model was the Exponential Smoothing + Decomposition based on the SMAPE and the SARIMA based on the RMSE.

**Table 2:** Accuracy measures (RMSE and SMAPE) for the model forecast of each of the five considered models, in the data from the three monitoring stations, BH1, Itabira4, and S.J.daLapa2.

	BH1		Itabira4		S.J.daLapa2	
	RMSE	SMAPE	RMSE	SMAPE	RMSE	SMAPE
Seasonal Naive	11.24	0.58	9.85	0.57	34.31	0.41
Naive + Dec	8.74	0.35	7.11	0.32	30.10	0.43
Exp Smoothing + Dec	9.06	0.36	7.11	0.30	26.45	0.28
SARIMA	8.23	0.35	7.02	0.31	31.05	0.35
NNETAR	10.41	0.39	11.52	0.52	47.00	0.65
NNETAR + Dec	8.64	0.34	9.39	0.47	33.34	0.50

## Conclusion

The approach presented in this paper provided a spatio-temporal and descriptive analysis of the behavior of the ${\text {PM}}_{10}$ concentrations in 29 monitoring stations in the Brazilian state of Minas Gerais. The use of boxplots per hour of the day, per day of the month, per month of the year, and per year, allowed us to find specific trends and patterns. Besides the seasonal patterns, an increase in the ${\text {PM}}_{10}$ concentrations was visible in BH1 from 2018 and especially at the end of 2019. S.J.daLapa2 is the monitoring station with the highest average concentration of ${\text {PM}}_{10}$, likely due to lime and crushed stone factories located in the region, with an average concentration of 49.9 $\upmu$g/m$^3$ against 25.37 $\upmu$g/m$^3$ and 22.13 $\upmu$g/m$^3$ in BH1 and Itabira4, respectively.

For the modeling and forecast part of the paper, six standard and more advanced models for time series were considered, as well as three monitoring stations: BH1, the capital city of the Brazilian state of Minas Gerais, and the monitoring stations with the lowest and highest average ${\text {PM}}_{10}$ concentration levels. The overall best models for model fit were the NNETAR and NNETAR+decomposition, and the overall best models for forecasting were the SARIMA and Exponential Smoothing + decomposition. This difference could be because of the small difference in RMSE and SMAPE between several models in the model fit.

Although the methodologies used in this study have been widely used for time series forecasting in general and to forecast ${\text {PM}}_{10}$ concentrations in particular, no comprehensive study including all monitoring stations in the Brazilian state of Minas Gerais has been made. Therefore the results and analyses presented in this paper, both in terms of model fit to better understand the historical behavior and of model forecast to predict the coming hours and days are of great potential relevance for local governments and policymakers to understand the dynamics of the ${\text {PM}}_{10}$ concentrations and take the necessary action to improve the environment and public health.

Some of the limitations of this study that can be considered as future working directions are: (1) the forecasting models discussed in this paper might not fully capture the whole signal in the data and others, e.g., based on deep learning^48,49^ and hybrid methods^50,51^, can be considered for all 29 monitoring stations in the Brazilian state of Minas Gerais to better understand the overall behavior; (2) the modeling and forecasting are based on univariate time series models and without geographical information, that can potentially be improved when considering multivariate and station-temporal models^52^; and (3) the influence of climate variables such as temperature, wind speed, radiation, and humidity, is not accessed in this paper, but their use might help to improve the forecasts and the spatio-temporal modeling approach as covariates.

## Supplementary Information

Supplementary Information.

## Data Availability

The data is available as supplementary material for this paper.
