# Supplementary material for: Spatio-temporal visualization and forecasting of PM10 in the Brazilian state of Minas Gerais
Source: Sci Rep. 2023 Feb 25;13:3269. doi: 10.1038/s41598-023-30365-w (PMC9968292; doi:10.1038/s41598-023-30365-w)
Supplement: Supplementary file 1 — Supplementary Information. [file 41598_2023_30365_MOESM1_ESM.pdf]

Spatio-temporal visualization and forecasting of PM_{10} in the Brazilian state of Minas Gerais

Kim Leone Souza da Silva[1], Javier Linkolk López-Gonzales[2,*], Josue E. Turpo-Chaparro[2], Esteban Tocto-Cano[3], and Paulo Canas Rodrigues[1]

[1]Department of Statistics, Federal University of Bahia, Salvador, Brazil
[2]UPG Ingeniería y Arquitectura, Escuela de Posgrado, Universidad Peruana Unión, Lima, Peru
[3]Facultad de Ingeniería y Arquitectura, Universidad Peruana Unión, Lima, Peru

Table 1S. Detailed information for each monitoring station, including, code, name of the station, company responsible for the monitoring station, longitude, latitude, and the rate of missing values.

Name	Name of station	Responsible Company	Longitude	Latitude	Rate of missing values
Betim1	Alterosa	Refinaria Gabriel Passos	44d09’39.06"W	19d56’51.18"S	0.1673
Betim2	Centro Administrativo Betim	Refinaria Gabriel Passos	44d12’26.00"W	19d58’07.98"S	0.0877
Betim3	Petrovale	Refinaria Gabriel Passos	44d06’40.05"W	19d59’39.93"S	0.1216
BH1	Centro Av. do Contorno	Refinaria Gabriel Passos	43d56’08.87"W	19d54’48.02"S	0.0438
BH2	Delegacia Amazonas	V&M do Brasil S.A	43d59’41.50"W	19d56’14.90"S	0.2250
Brumadinho2	Comunidade do Feijão	Vale S.A	44d06’32.19"W	20d08’09.13"S	0.2492
C. Fabriciano	Senac - (Vale do Aço)	Aperam Inox S.A	42d37’38.00"W	19d31’49.00"S	0.1119
Contagem	Cidade Industrial	V&M do Brasil S.A	44d01’54.27"W	19d57’38.07"S	0.2709
Ibirité1	Cascata	Refinaria Gabriel Passos	44d05’10.73"W	19d59’15.31"S	0.1019
Ibirité2	Piratininga (Ibiritermo)	Refinaria Gabriel Passos	44d03’34.89"W	20d00’14.27"S	0.0471
Ipatinga1	Bom Retiro	Usiminas	42d33’25.28"W	19d30’34.55"S	0.0877
Ipatinga2	Cariru	Usiminas	42d31’43.46"W	19d29’28.92"S	0.0929
Ipatinga3	Cidade Nobre	Usiminas	42d33’36.74"W	19d27’40.22"S	0.0745
Itabira2	Félix (EAMA 31)	Vale S.A	43d14’14.68"W	19d39’15.00"S	0.1909
Itabira3	Major Lage (EAMA 21)	Vale S.A	43d14’13.02"W	19d38’08.09"S	0.0560
Itabira4	Panorama (EAMA 41)	Vale S.A	43d13’19.10"W	19d38’04.10"S	0.0885
Itabira5	Pará (EAMA 11)	Vale S.A	43d13’50.85"W	19d37’10.00"S	0.1924
Paracatu1	Clube da União	Kinross Gold Corporation	46d53’10.86"W	17d12’56.88"S	0.2263
Paracatu2	Copasa	Kinross Gold Corporation	46d52’27.36"W	17d12’19.74"S	0.2467
Paracatu3	Lagoa Trindade Rodrigues	Kinross Gold Corporation	46d49’58.50"W	17d09’01.47"S	0.1642
Paracatu4	São Domingos	Kinross Gold Corporation	46d52’27.36"W	17d11’53.82"S	0.1746
Paracatu5	Sérgio Ulhoa	Kinross Gold Corporation	46d52’30.60"W	17d13’28.44"S	0.1262
S.J.daLapa1	Célvia (Vespasiano)	Belocal	43d58’07.83"W	19d42’45.01"S	0.2041
S.J.daLapa2	Centro (Delegacia)	Belocal	43d57’36.70"W	19d42’00.54"S	0.1703
S.J.daLapa3	Jardim Encantado	ICAL	43d56’17.33"W	19d41’49.21"S	0.2114
S.J.daLapa4	Escola Municipal Filinha Gama	ICAL	43d57’50.73"W	19d42’47.45"S	0.3371
Timóteo1	Cecília Meireles	Aperam Inox S.A	42d39’23.00"W	19d32’47.00"S	0.2533
Timóteo2	Escola Sementinha	Aperam Inox S.A	42d40’16.00"W	19d32’12.00"S	0.0908
Timóteo3	Hospital Vital Brazil	Aperam Inox S.A	42d38’33.00"W	19d32’59.00"S	0.1017

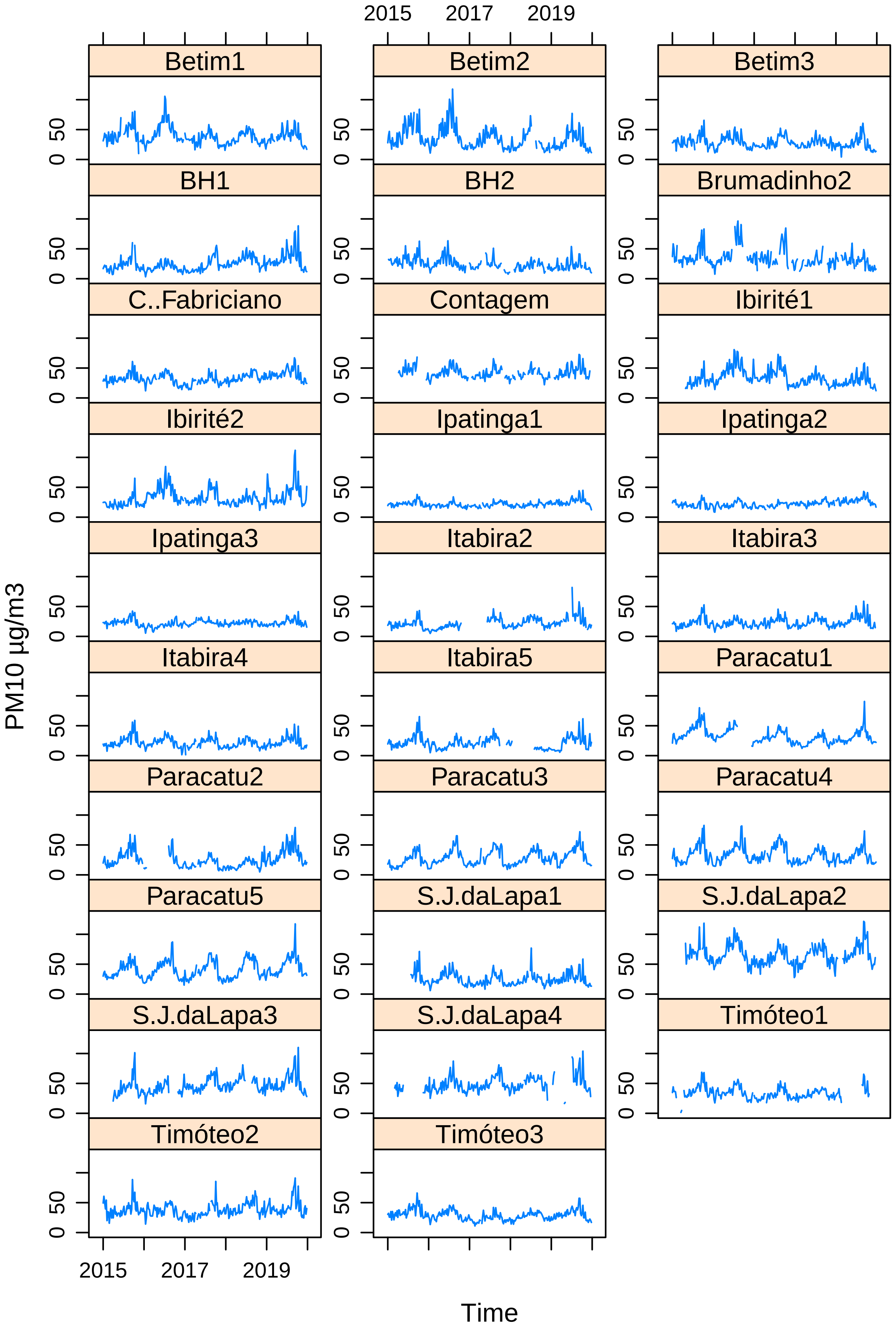

Figure 1S: Time series of the weekly average PM_{10} (in $\mu g/m^3$) concentrations for all 29 monitoring stations.

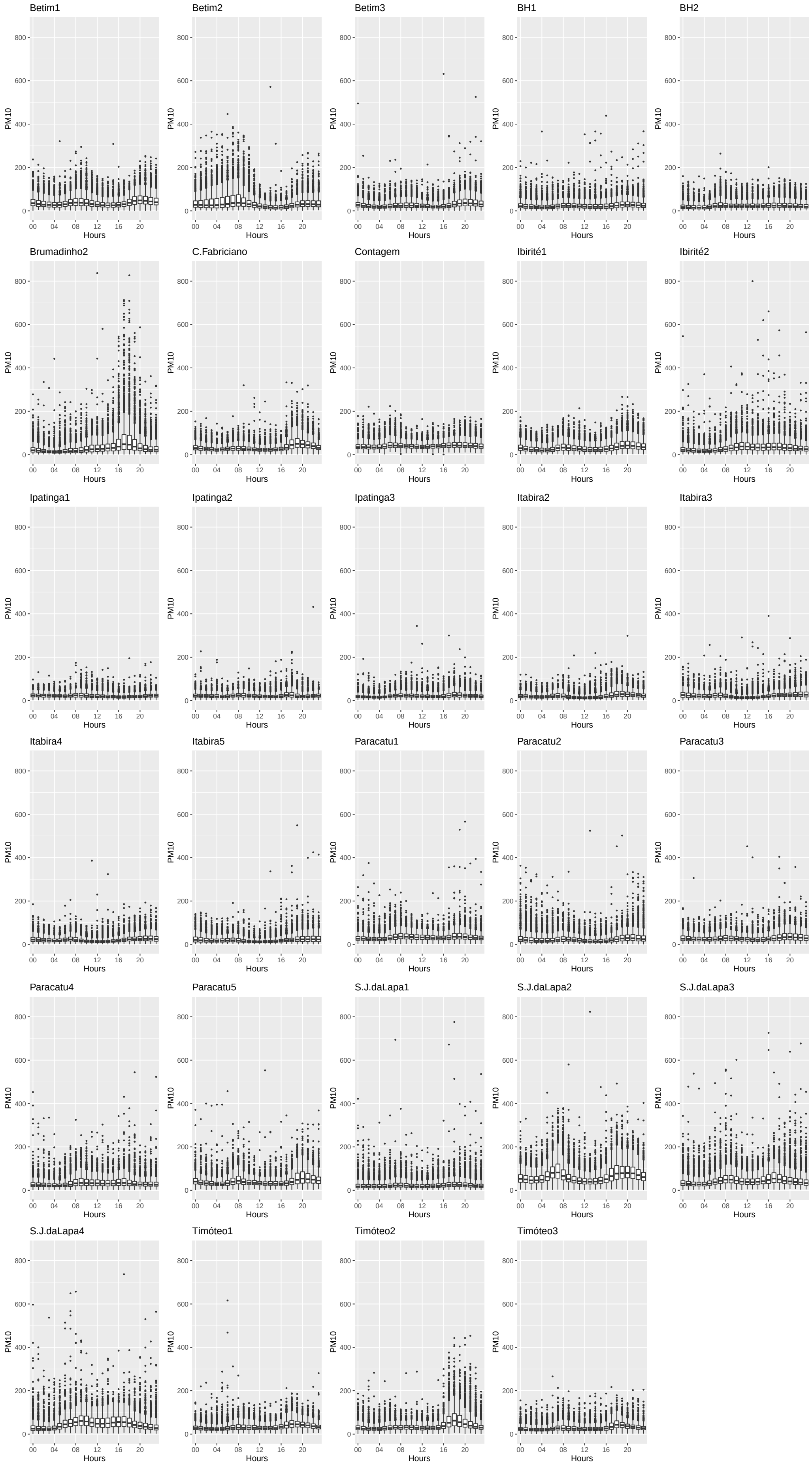

Figure 2S: Boxplots of the PM_{10} concentrations (in $\mu g/m^3$) per hour of the day for all 29 monitoring stations (e.g., the first boxplot in each plot is based on all PM_{10} concentrations observed between midnight and 1 am). The plots follow the same order as in Table 1S from left to right and from top to bottom.

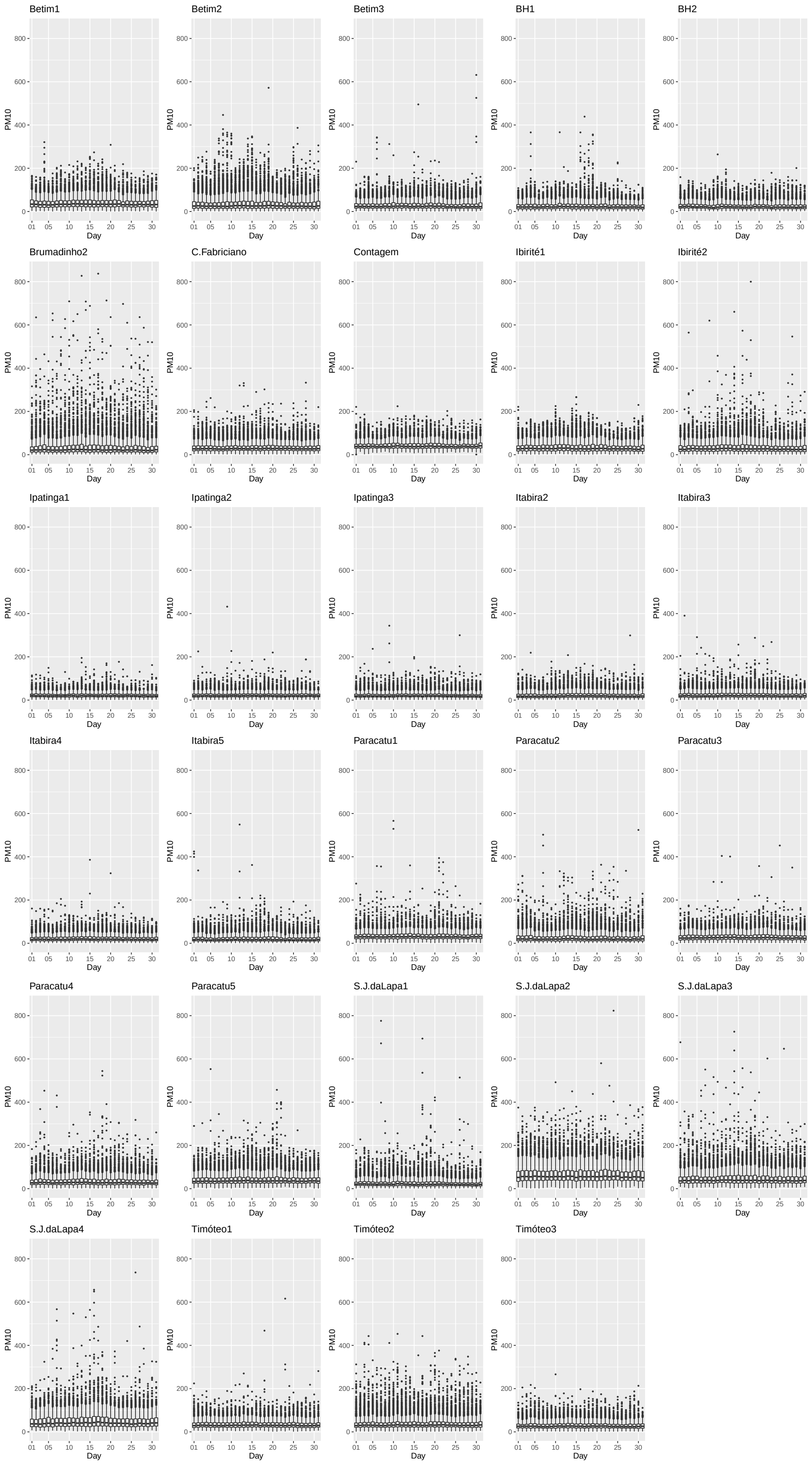

Figure 3S: Boxplots of the PM_{10} concentrations (in $\mu g/m^3$) per day of the month for all 29 monitoring stations (e.g., the first boxplot in each plot is based on all PM_{10} concentrations observed on the first day of the month). The plots follow the same order as in Table 1S from left to right and from top to bottom.

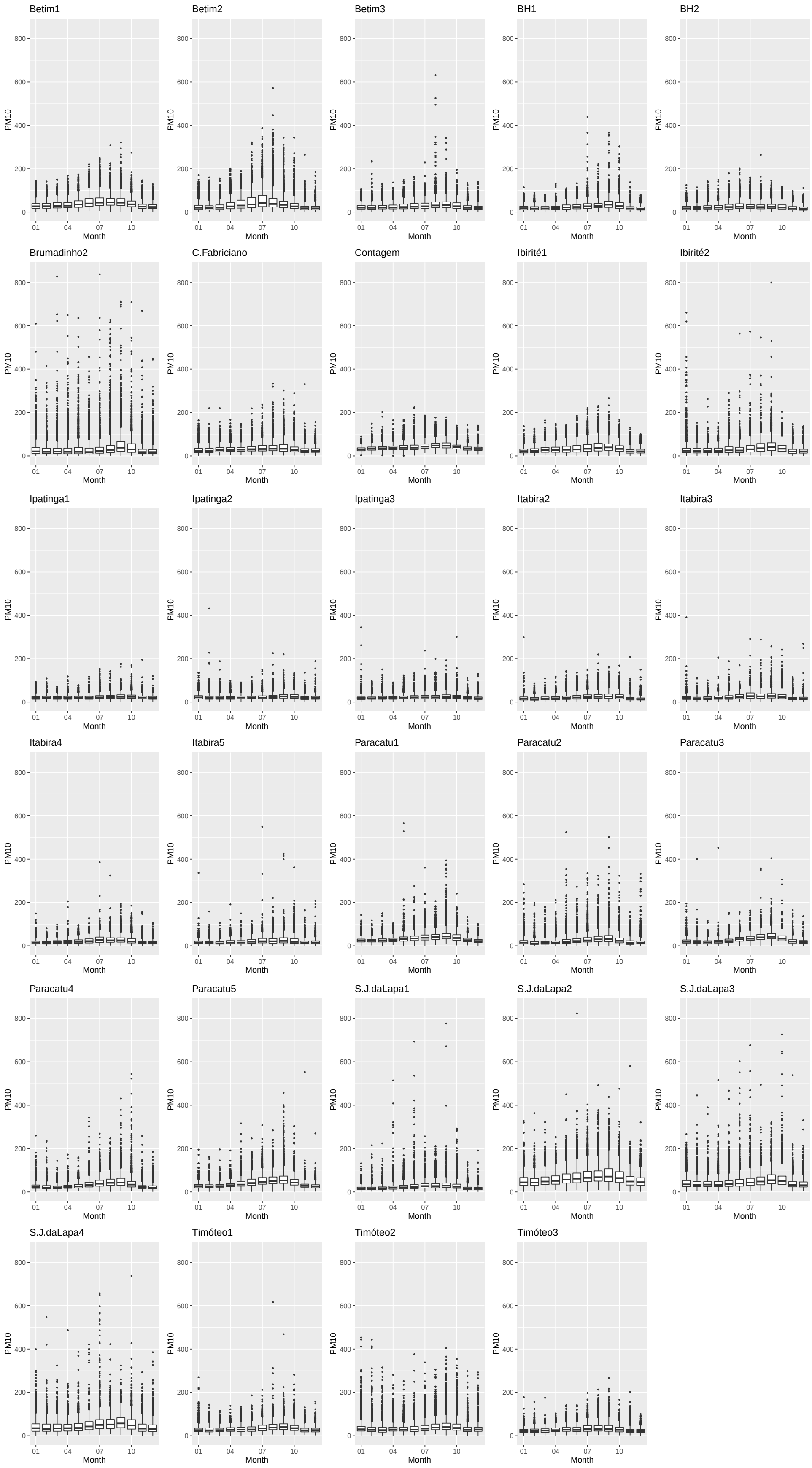

Figure 4S: Boxplots of the PM_{10} concentrations (in $\mu g/m^3$) per month of the year for all 29 monitoring stations (e.g., the first boxplot in each plot is based on all PM_{10} concentrations observed on January). The plots follow the same order as in Table 1S from left to right and from top to bottom.

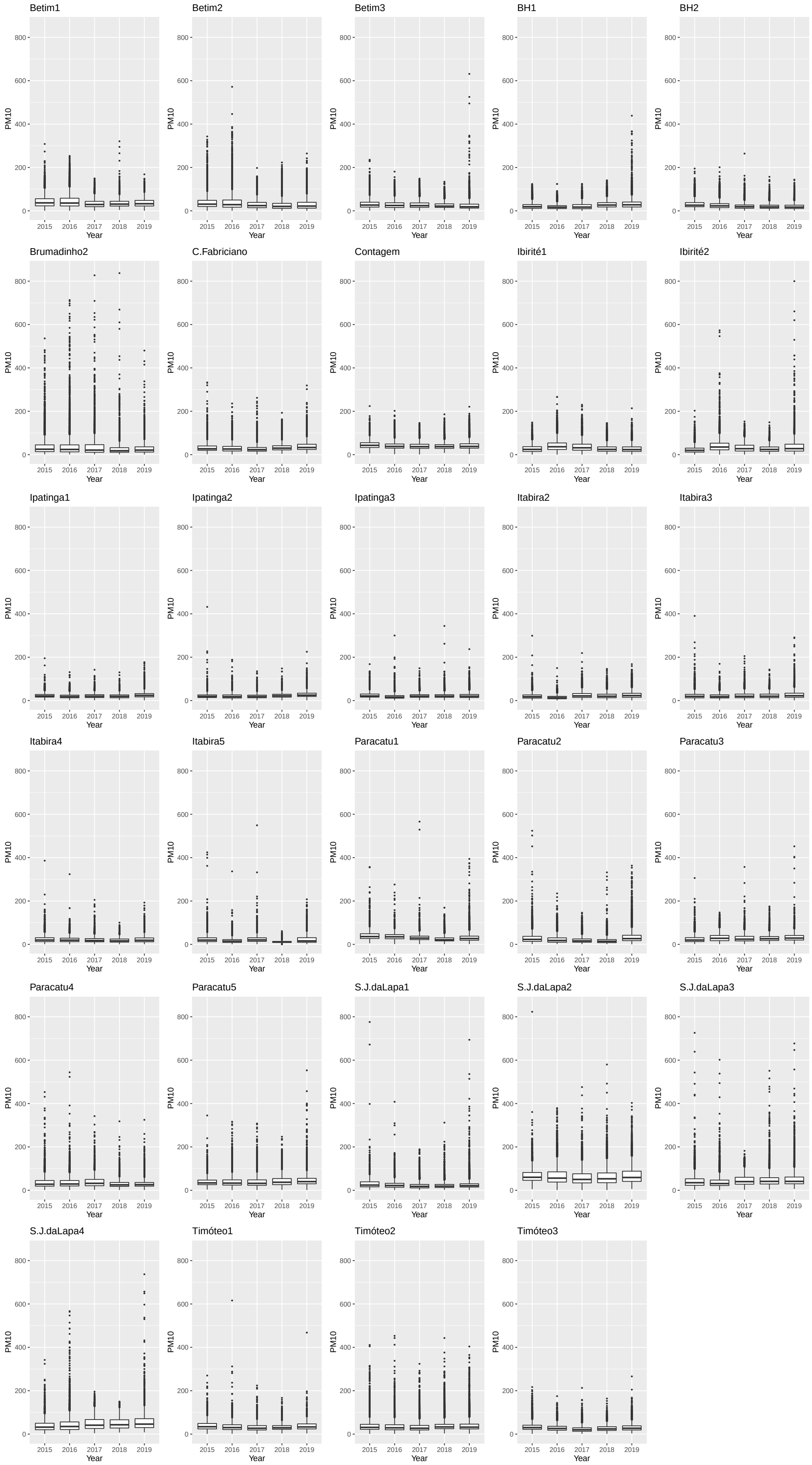

Figure 5S: Boxplots of the PM_{10} concentrations (in $\mu g/m^3$) per year for all 29 monitoring stations (e.g., the first boxplot in each plot is based on all PM_{10} concentrations observed in 2015). The plots follow the same order as in Table 1S from left to right and from top to bottom.

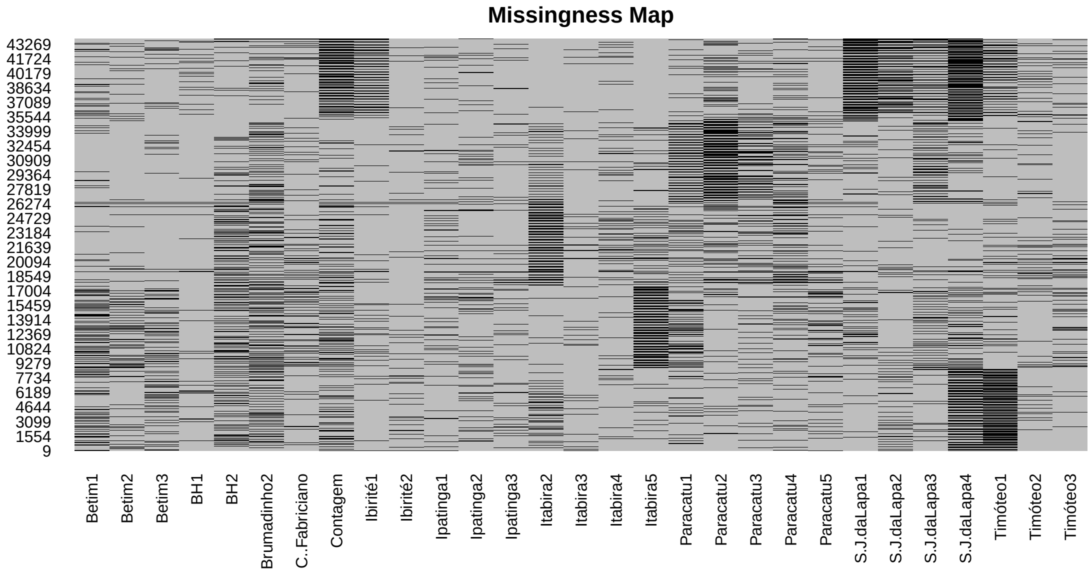

Figure 6S: Heat map with the position of missing values in all considered monitoring stations. The vertical axis shows the hourly observations, and the horizontal axis the 29 monitoring stations. Grey marks indicate that the data point was observed, and black marks indicate the missing values.
